# Development and Implementation of a Real-time Bundle-adherence Dashboard for Central Line-associated Bloodstream Infections

**DOI:** 10.1097/pq9.0000000000000431

**Published:** 2021-06-23

**Authors:** Augustine Chemparathy, Martin G. Seneviratne, Andrew Ward, Simran Mirchandani, Ron Li, Roshni Mathew, Matthew Wood, Andrew Y. Shin, Lane F. Donnelly, David Scheinker, Grace M. Lee

**Affiliations:** From the * Department of Management Science and Engineering, Stanford University, Stanford, Calif.; † Department of Biomedical Informatics, Stanford University, Stanford, Calif.; ‡ Department of Electrical Engineering, Stanford University School of Engineering, Stanford, Calif.; § Center for Biomedical Informatics Research, Stanford University School of Medicine, Stanford, Calif.; ¶ Lucile Packard Children’s Hospital, Stanford University, Stanford, Calif.

## Abstract

**Introduction:**

Central line-associated bloodstream infections (CLABSIs) are the most common hospital-acquired infection in pediatric patients. High adherence to the CLABSI bundle mitigates CLABSIs. At our institution, there did not exist a hospital-wide system to measure bundle-adherence. We developed an electronic dashboard to monitor CLABSI bundle-adherence across the hospital and in real time.

**Methods:**

Institutional stakeholders and areas of opportunity were identified through interviews and data analyses. We created a data pipeline to pull adherence data from twice-daily bundle checks and populate a dashboard in the electronic health record. The dashboard was developed to allow visualization of overall and individual element bundle-adherence across units. Monthly dashboard accesses and element-level bundle-adherence were recorded, and the nursing staff’s feedback about the dashboard was obtained.

**Results:**

Following deployment in September 2018, the dashboard was primarily accessed by quality improvement, clinical effectiveness and analytics, and infection prevention and control. Quality improvement and infection prevention and control specialists presented dashboard data at improvement meetings to inform unit-level accountability initiatives. All-element adherence across the hospital increased from 25% in September 2018 to 44% in December 2019, and average adherence to each bundle element increased between 2018 and 2019.

**Conclusions:**

CLABSI bundle-adherence, overall and by element, increased across the hospital following the deployment of a real-time electronic data dashboard. The dashboard enabled population-level surveillance of CLABSI bundle-adherence that informed bundle accountability initiatives. Data transparency enabled by electronic dashboards promises to be a useful tool for infectious disease control.

## INTRODUCTION

Central line-associated bloodstream infections (CLABSIs) are hospital-acquired infections responsible for significant morbidity and healthcare expenditure in pediatric populations.^1,2^ CLABSIs are the most common nosocomial infection in pediatric patients and have been estimated to extend a patient’s admission by 19 days and cost an average of over $55,000 per infection.^3,4^

One of the most successful CLABSI reduction strategies is the central line bundle—a set of best practices for inserting and maintaining central lines.^5,6^ Components of the maintenance bundle include daily review of line necessity, chlorhexidine gluconate (CHG) baths, scrubbing the access port, changing administration sets, and routine dressing changes. Bundle-adherence should be high to drive clinical outcomes; adherence to the CLABSI bundle greater than 95% is associated with a decrease in the CLABSI rate.^7^

Interviews with the nursing staff at our hospital revealed that bundle-adherence was inconsistent across clinical units. Some units would systematically not complete one or several bundle elements, causing their all-element adherence to fall to zero. An EHR audit estimated that across the hospital, all-element adherence was approximately 10% before the intervention. Reasons cited for nonadherence included clinical inappropriateness of a particular bundle element, lack of documentation for a particular bundle element, lack of time, and low prioritization. Some units cited special considerations that systematically caused them to omit some bundle elements; for instance, staff in the Hematology/Oncology unit deemed the daily review of line necessity not relevant as patients had lines in place on a long-term basis. Also, neonatal intensive care unit (NICU) practice was variable regarding CHG bathing due to concerns about use in preterm infants, so CHG bathing was not performed consistently.

In the past, our hospital relied on manual CLABSI bundle audits and reviews by each clinical unit to measure bundle-adherence. These reviews focused on accountability for individual bundle elements. This process had several drawbacks. Units differed in how bundle-adherence data were collected and who was responsible for collecting it. These data were collected through random auditing instead of a comprehensive audit of all patients with central lines. Last, each unit focused on specific bundle elements with the most significant adherence gaps rather than on all-element adherence.

The electronic health record (EHR) presents an opportunity to give clinical decision-makers greater transparency into real-time clinical quality metrics and key patient safety indicators across units.^8–10^ EHR-based quality dashboards have shown promise in improving care quality across various domains, including infection control,^11^ fall prevention,^12^ and prescription errors.^13^

To support hospital-wide CLABSI reduction efforts, we sought to develop a standard process to measure bundle-adherence across clinical units. This process leveraged real-time electronic data sources to create data visualization tools to support ongoing improvement efforts. Developing such a system requires significant effort and collaboration across teams of care providers, medical and operational leaders, and IS. However, no CLABSI-specific roadmap for how to achieve this was available. We believe that a detailed description of the primary challenges and innovations in developing such a system may reduce the difficulty of similar work for other institutions.

This report outlines the development, implementation, and use of an automated, hospital-wide CLABSI bundle-adherence system to enable nursing staff and infection control groups to track adherence rates with individual bundle elements between units and over time. We focus on the key stakeholders and processes, the most critical components of data analysis, and lessons learned from the dashboard’s deployment.

## METHODS

Lucile Packard Children’s Hospital (LPCH) is a 395-bed academic, freestanding children’s hospital. The proposed intervention was an automated, hospital-wide bundle-adherence system leveraging data from the commercial EHR Epic (Epic Systems, Verona, Wis.). The primary elements of this adherence system are the operational considerations of its planned use and the technical deployment of the information necessary via an EHR dashboard.

### Operational Design

The hospital’s CLABSI reduction workgroup developed a plan to identify stakeholders in charge of monitoring bundle-adherence, seek feedback from these stakeholders about the system’s proposed use and functionality, and design a mechanism to evaluate the system’s usefulness after deployment.

The primary stakeholders of the proposed bundle-adherence system were (1) the Infection Prevention and Control (IPC) team, who defined the standards and definitions used to measure bundle elements; (2) clinical unit leaders, who helped identify where bundle-adherence information was presently documented and who could support accountability measures for bundle-adherence; and (3) the data analytics team, who enabled the extraction of real-time bundle-adherence data. An additional stakeholder was Systems Utilization Research for Stanford Medicine, an interdisciplinary group of physicians and Stanford School of Engineering students enabling the development of the dashboard and integrating real-time data streams.

The development of a dashboard with visual representations of bundle-adherence rates across units and bundle elements was proposed. We designed the dashboard in consultation with a multidisciplinary group comprising physicians, nurses, data analytics and quality specialists, and engineers. To evaluate the dashboard, we recorded electronic dashboard accesses and all-element adherence data for each unit after deployment. Workgroup leaders collected feedback about the electronic dashboard from unit leaders after deployment.

### Dashboard Development and Deployment

**Fig. 1.**
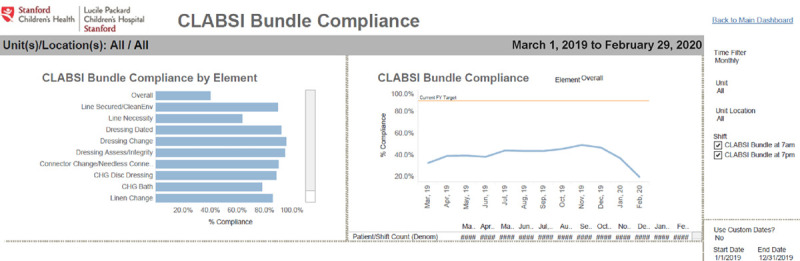
Screenshot of the interactive dashboard to track CLABSI bundle-adherence between units, including adherence rates for the complete bundle and individual elements. ICN, intensive care nursery; PCU, patient care unit.

The workgroup data analytics team developed a data pipeline to pull bundle-adherence data from SAP Webi (SAP, Walldorf, Germany), postprocess it in R (R Foundation for Statistical Computing, Vienna),^14^ and import it into Tableau (Tableau Software, Seattle, Wash.) to generate a dashboard. Adherence calculations were based on data entered by nursing staff into the EHR during bundle checks, which routinely occurred twice daily (morning and evening). We added a per-shift attestation to the EHR during dashboard implementation to affirm that nursing staff had completed the bundle-adherence checklist elements for each patient with a central line. Unit-level bundle-adherence rates were calculated as the percentage of patients for whom all bundle elements were in compliance at the time of a bundle check, averaged across a customizable time window. Adherence rates for each bundle element were calculated as the average percentage of patients with that specific element satisfied at the bundle check. Adherence calculations took into account the different frequencies at which bundle elements must be repeated. For instance, line necessity is documented daily, whereas dressing changes are documented weekly. Thus, the lookback periods varied for each bundle component. Users can use an interactive slider to specify a lookback time window and select a specific unit (eg, cardiac ICU) to visualize the unit’s individual element breakdown (Fig. 1).

## RESULTS

### Dashboard Deployment

In April 2018, the data analytics and Systems Utilization Research for Stanford Medicine teams developed a dashboard prototype. For 3 months, the data analytics team updated the dashboard with manual (nonautomated) data pulls, presented to stakeholders, and modified the dashboard based on stakeholder feedback. Following consultation with information services (IS) and hospital leadership, the dashboard was deployed in September 2018 through a Tableau server using data pulled automatically from the EHR.

The rollout of the dashboard led to the identification of challenges and focused interventions. Initially, the documentation of line necessity elements was not accurately pulled in from the various areas in the EHR where it was being documented by nursing staff. Hospital IS solved this problem by accounting for these additional documentation sites when pulling real-time bundle-adherence data. Also, low rates of adherence to CHG bathing were identified in the NICU and oncology unit. The NICU created guidelines to clarify the use of CHG bathing only for pediatric patients older than 48 weeks corrected gestational age, and electronic documentation was modified to reflect appropriate adherence based on gestational age. In the oncology unit, a targeted intervention was initiated to identify and reduce instances when “allergy” was inappropriately documented in the EHR as a contraindication for CHG bathing. Nursing staff also initiated a pilot study of an alternate CHG product that they felt would increase CHG bathing adherence by having a more comfortable feeling on patients’ skin.

### Usage Metrics

**Fig. 2.**
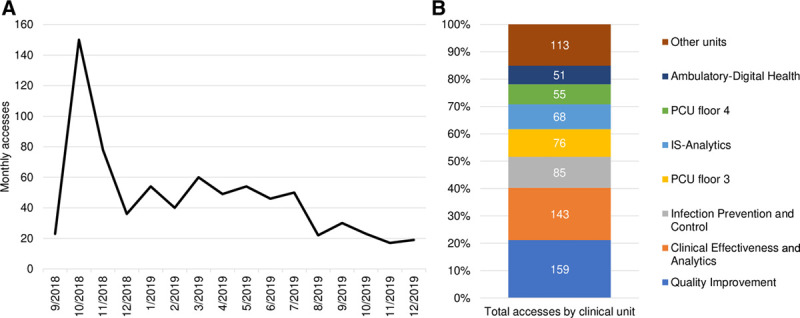
Accesses of the CLABSI bundle-adherence dashboard. A, Hospital-wide monthly accesses between September 2018 and December 2019. B, Total accesses between September 2018 and December 2019, broken down by clinical unit. Units with fewer than twenty accesses during this period are grouped in “Other units.” PCU, patient care unit.

The dashboard was accessed 750 times between September 2018 and December 2019. Monthly usage peaked at 150 accesses in October 2018 and remained between 15 and 30 accesses per month between August 2019 and December 2019 (Fig. 2A). The departments with the highest dashboard usage were quality improvement (QI) (159 accesses), clinical effectiveness and analytics (143 accesses), and IPC (85 accesses) (Fig. 2B). The dashboard also had high usage in 2 different acute care medical units. One acute care unit recorded 76 accesses and the other 55.

### Bundle-adherence Metrics

**Fig. 3.**
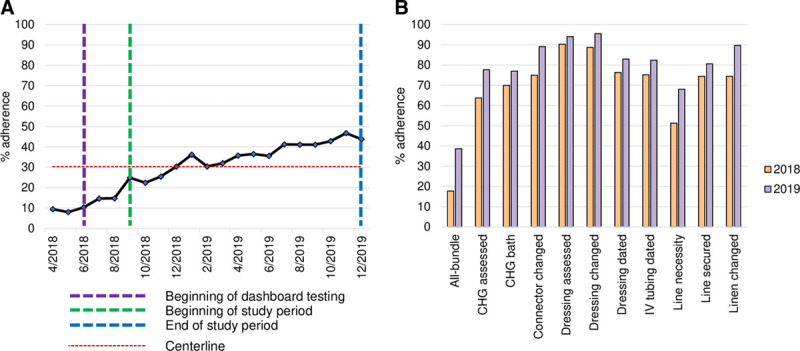
Adherence to the CLABSI bundle. A, Average hospital-wide adherence to all bundle units between September 2018 and December 2019. B, Average hospital-wide adherence for individual bundle elements in 2018 and 2019.

**Fig. 4.**
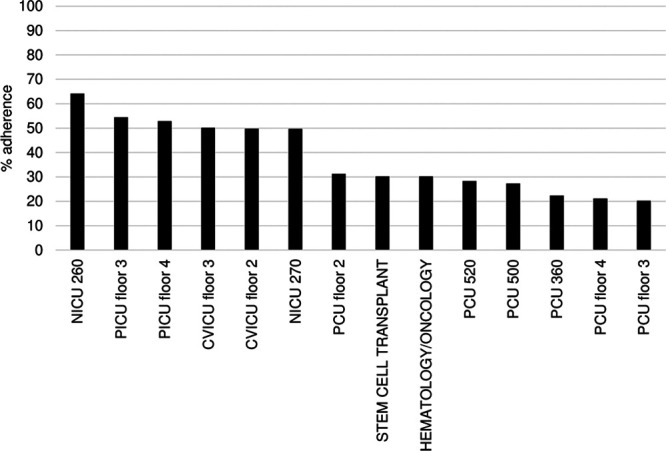
Average adherence to all bundle units by clinical unit, recorded between January 2019 and December 2019. PCU, patient care unit.

The average all-element adherence across the hospital increased from 25% in September 2018 to 44% in December 2019 (Fig. 3A). Moreover, average adherence for each bundle element increased between 2018 and 2019 (Fig. 3B). Unit-level data collected between January 2019 and December 2019 demonstrated that all-element adherence varied across clinical units (Fig. 4). Adherence was highest in the following units: NICU 260 (64%), the pediatric intensive care unit (PICU) floor 3 (54%), PICU floor 4 (53%), and cardiovascular intensive care unit (CVICU) (50%). Despite the high number of accesses of the bundle dashboard by staff in the acute care units, these units did not demonstrate higher all-element adherence than other units.

### Feedback from the Nursing Staff

Following the hospital-wide launch of the bundle-adherence dashboard, feedback reflected variable adoption across hospital staff groups. IPC specialists and QI leads have primarily utilized the dashboard to understand unit-level bundle-adherence. Data were routinely presented at local improvement meetings, including physician leaders, nursing leaders, QI specialists, and IPC staff. These unit-based meetings facilitated engagement on improvements between IPC, QI, unit-level leaders, and other front-line staff. The bundle-adherence dashboard is used consistently by the physician and nurse leaders of the PICU and CVICU to understand opportunities for improvement. However, the NICU, Hematology/Oncology, and stem cell transplant units use the dashboard less frequently. These latter units have previously established processes for bundle rounds and trust them to be more accurate, resulting in a hesitancy to utilize the dashboard. For instance, the NICU uses real-time coaching on bedside rounding for all patients with central lines and prefers this to the automated process.

### CLABSI Rate

**Fig. 5.**
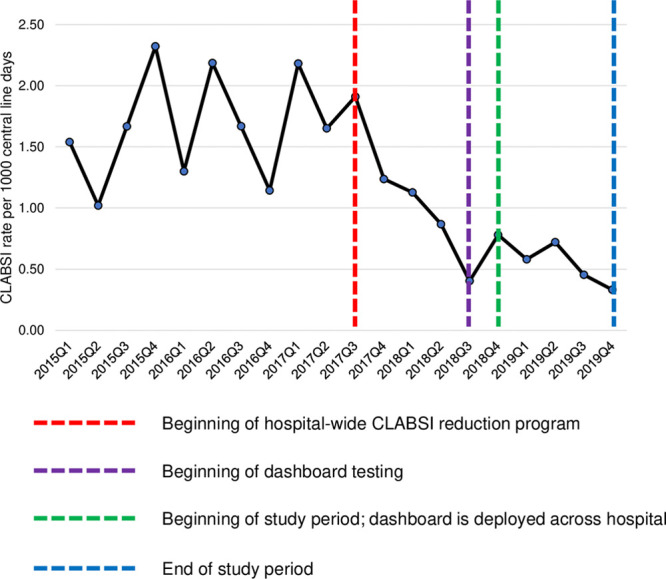
Hospital-wide CLABSI rate trend beginning in 2015 Q1 and extending until the end of the study period in 2019 Q4.

We implemented the bundle-adherence dashboard in parallel with several simultaneous hospital-wide practice changes targeted at CLABSI reduction. The 3 key drivers of this initiative were (1) data transparency through the development of the CLABSI bundle-adherence dashboard; (2) standardization of practice and equipment; and (3) accountability. CLABSI rates across the hospital declined substantially as visibility and accountability became the new norm. The detailed analyses of EHR data on central line usage and CLABSI rates also led to insights about national criteria for measuring CLABSI rates.^15,16^ Following the rollout of the hospital-wide interventions, the CLABSI rate at LPCH declined from 1.0–2.3 CLABSIs per 1,000 line days between January 2015 and September 2017 to 0.4–0.7 CLABSIs per 1,000 line days between July 2018 and June 2019, constituting a statistically significant decrease (*P* = 0.001).^6^ During the study period, the hospital-wide CLABSI rate remained stable, changing from 0.82 CLABSIs per 1,000 line days in 9/2018 to 0.65 CLABSIs per 1,000 line days in December 2019 (Fig. 5).

## DISCUSSION

This report describes the development and rollout of a dashboard to track adherence rates with the central line maintenance bundle in real-time across all units in a pediatric academic medical center. This dashboard’s automated nature enables decision-makers to carry out real-time, population-level surveillance of bundle-adherence rather than a retrospective or sampling-based surveillance. Presenting live data to both the infection control team and front-line clinicians enables feedback so that clinical units know their level of bundle-adherence and can identify opportunities for improvement.

Collaboration between interdisciplinary stakeholders was key to the development of the dashboard. Clinical insights from unit leaders and IPC informed the development of a data visualization tool that integrates real-time data streams from hospital IS. The collaborative design stage was essential to ensure that the resulting tool was relevant and useful for stakeholders.

Users in IPC and QI primarily accessed the electronic dashboard. Although unit leads in the PICU and CVICU indicated that the dashboard was used consistently in these units, the number of dashboard views by users from these units was low. This observation may indicate that the unit leads depended on dashboard data relayed by IPC and QI leads instead of directly accessing the dashboard. The all-element adherence rate was highest in the NICU, PICU, and CVICU. Whereas the PICU and CVICU clinical leadership reported consistent dashboard use, the NICU used a custom process to ensure bundle accountability.

The hematology/oncology and stem cell transplantation units also had pre-existing, labor-intensive processes to monitor unit-wide bundle adherence. Staff in these units accessed the electronic dashboard relatively few times, which may be due to the perception that their patients were unique and low perceived applicability of certain bundle elements, such as daily line necessity checks or CHG bathing. Moreover, these units experience CLABSIs attributable to mucosal barrier injury and neutropenia, which may lead to the perception that intrinsic patient characteristics (ie, neutropenia, mucositis, etc.) are more important causes of CLABSI than lack of adherence to central line bundles in these units. CLABSI is a complex problem, and the degree of success of an intervention depends on each unit’s patient population, existing processes, perceived utility of a new tool, and operational culture.

The use of all-element bundle-adherence as a quality metric results in challenges in interpretation. The omission of just 1 bundle element causes all-element bundle-adherence to be zero. Lack of documentation of individual elements such as line necessity can lower all-or-none adherence rates despite adherence to all other bundle elements. Nonetheless, electronic dashboards represent an opportunity to improve adherence consistently on individual bundle elements and ultimately on all bundle elements.

The dashboard described here has similarities and differences relative to previous work in this space. At our institution, a patient-level CLABSI bundle checklist and unit-level CLABSI dashboard were deployed in the PICU in 2010 and showed a significant CLABSI rate decrease post-intervention.^17^ However, this dashboard was not scaled across all units. Its unit-level view was restricted to a list of visual signals (green, yellow, or red), indicating whether all bundle elements had been completed for each patient.

Another previous study implemented an EHR checklist for central line insertion bundle elements and postinsertion verification of line necessity.^18^ The EHR automatically generated a daily procedure note for the physician to fill out and gave relevant feedback on missing bundle elements. This intervention resulted in improved documentation compliance and quality of CLABSI tracking data. Similar to the PICU dashboard at our hospital, this intervention provided patient-level guidance to front-line clinicians. However, this study did not incorporate a unit-level bundle-adherence summary.

An additional study implemented a patient-level, real-time EHR dashboard to monitor compliance with a ventilator-associated pneumonia bundle in the surgical ICU.^11^ This dashboard uses visual signals (green, yellow, or red) to indicate each bundle element’s status for the patient. The bundle-adherence for each patient was reviewed during twice-daily rounding, and clinical leadership received daily reports on adherence. Following the dashboard’s rollout, the unit observed a statistically significant increase in bundle-adherence and decreased ventilator-associated pneumonia rate. This study is similar to ours in its use of real-time EHR data to inform bundle accountability efforts by unit-level leadership. Our study extends upon this by enabling clinical leaders to carry out unit-to-unit comparisons at the bundle element and all-element levels.

A limitation of this study is the challenge of ensuring accurate EHR entries for each daily bundle element. We did not verify the accuracy of nursing documentation or track adherence to data entry in this project. Nursing documentation for bundle elements is part of routine patient care, and the data elements for our dashboard are derived from the nursing flowsheet. However, nurses may document essential information in multiple places, and practices may vary by unit. We overcame this limitation by working with IS to pull in data from multiple locations, though future interventions could focus on streamlining documentation practices and workflow to minimize variability.

Digital dashboards are also susceptible to dynamic variability in documentation practices. Following the study period, in December 2019–February 2020, there was a recorded drop in the all-element bundle adherence without a corresponding increase in CLABSI rates. Although contributions to the decrease in all-element bundle adherence are likely multifactorial, we postulate high clinical volumes during those months may have impacted documentation practices, resulting in lower all-or-none adherence rates due to lack of documentation of just 1 or 2 bundle elements.

One additional limitation is the inability to track relative usage between the new hospital-wide dashboard and pre-existing patient-level checklists. The dashboard provided aggregate data and served different audiences—QI and IPC used hospital-level data, and nursing leaders used unit-level data. In contrast, individual nurses at the bedside can access patient-level bundle elements to complete tasks on their shift. We did not collect data from nursing staff regarding their comparative use of the unit-level and patient-level dashboards during dashboard evaluation.

A recent literature review of visualization dashboards in the EHR suggests that dashboards have the potential to streamline data collection, improve user satisfaction, and increase safety by enabling appropriate clinical decision-making with fewer medical errors.^16^ Moreover, widespread business intelligence tools such as Tableau, Qlik (Qlik, King of Prussia, Pa.), and native EHR packages have simplified the development of visualization dashboards on top of the EHR, making it easier for health systems to build customized quality dashboards. These trends promise to drive the development and adoption of real-time data dashboards, such as the one described in this study.

Customizable dashboard packages can be readily adapted to provide transparency around bundle-adherence rates between hospital units and over time. Data transparency stands to be an increasingly important driver of infectious disease control.

## DISCLOSURE

M.G.S. is currently an employee of Google and S.M. is currently an employee of Lyft. This work was completed while they were students at Stanford. The other authors have no financial interest to declare in relation to the content of this article.
